# Assessment of online newspapers’ framing directions of COVID-19 outbreak data in Nigeria

**DOI:** 10.1016/j.dib.2025.111985

**Published:** 2025-08-14

**Authors:** Fola Bamigbolayin-Afolabi, Kehinde Oyesomi, Lanre Amodu, Nelson Okorie, Evaristus Adesina, Gloria Omale

**Affiliations:** a Department of Mass Communication, Covenant University, Ota, Ogun State, Nigeria; b Pan-Atlantic University, Lagos State, Nigeria; c Indigenous Language Media in Africa Entity, Northwest University. South Africa; d Federal University of Technology, Minna, Niger State, Nigeria

**Keywords:** Assessment, COVID-19, Media framing, Online newspaper, Outbreak, Pandemic

## Abstract

Literature is scarce on an explorati on of the peculiar mix of the particular directions of framing employed in this study-critical framing, distance framing, empathy framing and support framing. The study sought to compare COVID-19 crisis frame directions in 5 selected Nigerian newspapers. The framing theory was employed as the theoretical framework for this study. A quantitative content analysis was the method adopted for the study. Content analysis data were gathered through the Wayback Machine website archive. Following a critical analysis of 4808 COVID-19 newspaper stories, framing direction findings indicated that Daily Post Newspaper stories largely used support framing, whereas Guardian and Punch Newspaper stories made extensive use of distance framing. Premium Times and Vanguard Newspaper stories used empathy framing. Therefore, this study recommends that in-house-style policies of media houses contain particular instructions on framing directions to reduce media bias and build audience trust. Health bodies (like the N.C.D.C. and the W.H.O.), health reporters and even health authorities can craft health stories in ways that the public can relate to. The stories can be told to encourage mitigation methods and to discourage misinformation, which builds trust in the readership. This study contributes to specific reportage strategies that could help future pandemic situations.

Specifications Table

**Table:** 

Subject	Social Sciences
Specific subject area	***Heath Communication, Online Journalism***
Type of data	***Primary Data (Tables)***
Data collection	***Quantitative Content Analysis via a Coding Guide.*** ***The Wayback Machine was used for the collection of Nigerian online newspaper Editions.*** *Inclusion criteria-* Nigerian online newspapers with keywords “Coronavirus” or “COVID-19”, Nigerian online newspapers that ran stories on “Coronavirus” or “COVID-19” and Nigerian online newspapers dedicated to news on “Coronavirus” or “COVID-19” in English Language alone. ***Exclusion criteria-*** Posts underneath the comments that are “Coronavirus” or “COVID-19” related, Strategy vs issue frames employed chiefly for political coverage- the frame type was irrelevant to this research since it is politics-based. This kind of framing has absolutely nothing to do with health, health communication, or COVID-19. This was left out of the coding manual. This is because the scope of this study does not cover the political implications of COVID-19, and it has no relevance to the objective of this study.
Data source location	Nigerian online Newspapers via WayBack Machine Internet Archive https://web.archive.org/ https://dailypost.ng/ https://guardian.ng/ https://www.premiumtimesng.com/ https://punchng.com/ https://www.vanguardngr.com/
Data accessibility	Bamigbolayin-Afolabi, Oluwafolafunmi; Oyesomi, Kehinde; Amodu, Lanre; Okorie, Nelson; Adesina, Evaristus; Eneh, Omale Gloria (2025), “AFOLABI FRAMING DIRECTIONS IN COVID-19 ARTICLES”, Mendeley Data, V1, doi: 10.17632/dkx4sszcjj.1 Repository name: Mendeley Data Data identification number: 10.17632/dkx4sszcjj.1 Direct URL to data: https://data.mendeley.com/datasets/dkx4sszcjj/1 Instructions for accessing these data: Open the Wayback Machine (https://web.archive.org)- Enter any of the newspaper websites in the search bar- Click on the year (2019, 2020 or 2021)- Access the Dates- Click on the last timestamp- Find the keywords “Coronavirus” or “COVID-19” in the articles (Ctrl + *F*)- Click on each article.
Related research article	None

## 1 Value of the Data

• The information will act as a foundation for additional studies within health communication in general.
• The findings on this data can open possibilities for future research studies on pandemic and epidemic communication.
• The information can be utilised in achieving SDG 3- Good health and well-being.

## 2 Background

Inadequate access to the right information for healthy living means poor health for the general public. One of the vital ways that information reaches the public is via the internet. This is the nexus between health communication and online newspapers [[1], [2], [3]]. Media framing is typically achieved through organizing media narratives and plays a significant part in the way information is perceived by the public. In spite of starting with traditional media, its use has been effortlessly extended to electronic media, showing how adaptable it is in different communication contexts [1]. Framing is one of the most influential methods the mass media use in covering health crises [[2], [3], [4]]. COVID-19 and newspaper framing have been studied a great deal.

Although there have been some studies that focused on newspapers and framing of COVID-19, none have touched on the particular set of directions of framing, as hinted by Chioma and Ojomo [5], which puts a premium on the 'tone' of the COVID-19 news reports. The particular directions of framing employed here are critical, distance, empathy, and support framing of COVID-19 news reports. Few studies have explored this particular set of these individual directions of the frames (critical, distance, empathy, and support) being used in media reports, particularly Nigerian online newspaper reports of COVID-19. This study thus attempts to analyse media framing directions in the COVID-19 news that was covered. The framing theory was employed as the theoretical framework for this study, and A quantitative content analysis was the method adopted.

## 3 Data Description

This study’s dataset contains Excel documents that have the headlines written verbatim, the dates of publication and the framing direction(s) that can be found in each of the articles' bodies Table 1, Table 2, Table 3 sourced from Bamigbolayin-Afolabi's mendeley repository [[6], [7]].

**Table 1:** Number of newspaper articles examined for each selected newspaper.

NAME OF NEWSPAPER	NEWSPAPER ARTICLES
DAILY POST	435
GUARDIAN	715
PREMIUM TIMES	916
PUNCH	1030
VANGUARD	1712
**TOTAL**	**4808**

**Table 2:** COVID-19 article distribution by framing directions and archival status in the 5 selected newspapers.

	DAILY POST	GUARDIAN	PREMIUM TIMES	PUNCH	VANGUARD	TOTAL
ARTICLES WITH FRAMING DIRECTIONS	5.9	14.5	20.3	21.7	37.6	100 %
ARTICLES WITH NO FRAMING DIRECTIONS	4.8	15.1	8.8	21	50.3	100 %
ARTICLES WITH NO COVID-19 STORY	11.8	7.4	28.8	26.4	25.6	100 %
NO WAYBACK MACHINE SNAPSHOTS	47.3	40	7.3	5.5	0	100 %
TOTAL	9	14.9	19.1	21.4	35.6	100 %
						*n* = 4808

**Table 3:** Distribution of framing directions of COVID-19 articles in the 5 selected newspapers.

	DAILY POST	GUARDIAN	PREMIUM TIMES	PUNCH	VANGUARD	TOTAL
CRITICAL FRAMING	7	14.4	16.3	21.4	40.8	100 %
DISTANCE FRAMING	3.7	17.5	20.1	24.8	33.9	100 %
EMPATHY FRAMING	66	11.3	24.2	22.1	35.9	100 %
SUPPORT FRAMING	79	12.7	26.9	16.4	36.1	100 %
NONE	4.8	15.1	8.8	21.0	50.3	100 %
NO COVID-19 STORY	11.8	7.4	28.8	26.4	25.6	100 %
NO WAYBACK SNAPSHOT	47.3	40	7.3	5.5	0	100 %
TOTAL	8.7	14.4	20.7	21.1	35.1	100 %
						*n* = 4808

## 4 Experimental Design, Materials and Methods

The subject of this study are Nigerian online newspapers, i.e., Nigerian newspaper publications providing news or general reports of events online (on digital media or websites). This study focused on online newspapers as they remained accessible during the pandemic lockdown, unlike printed media that was faced with distribution problems.

Quantitative content analysis was used in the research to explain patterns in communication content, establish correlations between the audience and the customized messages, and connect source characteristics to the messages they produce. Five Nigerian online dailies in 1570 editions were studied. Using online editions instead of print editions was made necessary by the unstable availability of hard copy newspapers in most African countries during the pandemic. The study's sampling frame comprised all the editions of newspapers published from December 31, 2019, through December 31, 2021—a two-year timeline purposed to follow the pandemic's trajectory. Study duration is limited from December 31st, 2019, through December 31st, 2021- to account for the initial wave of COVID-19 in Nigeria, 27th February 2020 to 24th October 2020, second wave, 25th October 2020 to 3rd April 2020, and third wave between June and November 2021 [8]. In content analysis, for this research, a multistage sampling method (two sampling processes)- purposive sampling at the first and systematic sampling at the second was employed.

In the purpose sampling, the research made use of the website ‘www.alexa.com’ to provide various forms of information regarding websites, including traffic data and search analytics [9]. Alexa has been operating since 1996, according to Manhas and Kaur [10], and has ``collected 4.5 billion pages from over 16 million sites.''. It is a site providing traffic statistics analysis of any website, such as traffic ranking and page views. Oladipupo [11], Ramadan, Osama, Zaher, Mansour, and El Sersi [12] and other scholars have demonstrated empirical evidence in the use of Alexa in other studies. The researcher typed the URL- ‘www.alexa.com’, typed 'Nigerian online newspapers' in the search box on the Alexa homepage and clicked the “search” button. For reliability testing, the Cronbach's Alpha Daily Post Critical Framing Analysis was 0.941, the Cohen's Kappa Daily Post Critical Framing Analysis was 0.888, the Cronbach's Alpha Daily Post Distance Framing Analysis was 0.775, the Cohen's Kappa Daily Post Distance Framing Analysis was 0.620, the Cronbach's Alpha Daily Post Empathy Framing Analysis was 0.937, the Cohen's Kappa Daily Post Empathy Framing Analysis was 0.881, the Cronbach's Alpha Daily Post Support Framing Analysis was 0.862, and the Cohen's Kappa Daily Post Support Framing Analysis was 0.758. The Cronbach's Alpha measure was above 0.70, while that of Cohen's Kappa was above 0.60, indicating that the coding guide was reliable.

## Limitations

Some of the dates (editions) in the Daily Post Newspaper had no Wayback Machine snapshots. This could happen as a result of technical issues, removal requests by the website’s owner, the website’s owner’s use of ‘the robots.txt’ file (preventing that specific edition from being archived), and poor crawling frequency-resulting in some pages or websites being archived more than others. Some of the dates (editions) in the selected newspapers had no COVID-19 story. The study had limitations in capturing editions (snapshots) that were missing from the Wayback Machine Internet Archive. This may subtly influence the total conclusions of this study. The time frame for the completion of this project was long, considering that almost 5000 articles were to be analysed using the Wayback machine in a short time. Uninterrupted internet access was needed for this study to explore the Wayback Machine; sometimes, it was unavailable. Wayback Machine was sometimes unavailable for use (Error 404).

## Ethics Statement

The authors have read and followed the ethical requirements for publication in Data in Brief and confirm that the current work does not involve human subjects, animal experiments, or any data collected from social media platforms. However, the data collected was from an online archive (https://web.archive.org/), and a clearance was obtained from the Covenant University Health Research Ethics Committee (CHREC). The following should also be noted:

1. Terms of Service (ToS): This study only used a small amount of data for content analysis. It follows the principles of Fair use because it is solely for academic use.
2. Copyright: Sources were properly cited, and this article did not republish full articles.
3. Privacy: It is not necessary to anonymise the data before sharing it because this is solely for academic use.
4. Scraping policies: For Wayback Machine and the news sites, scraping is allowed for non-commercial academic use.

## CRediT Author Statement

**Fola Bamigbolayin-Afolabi:** Conceptualization, Methodology, Writing; **Kehinde Oyesomi:** Supervision; **Lanre Amodu:** Supervision; **Nelson Okorie:** Writing – Review & Editing; **Evaristus Adesina:** Writing – Review & Editing; **Gloria Omale:** Writing – Review & Editing.

## Data Availability

Mendeley DataAssessment of online newspapers’ framing directions of covid-19 outbreak data in nigeria (Reference data). Mendeley DataPrimary (Reference data).
